# Construction of site selected phage library and characterization of anti-V3 scFvs from Indian clade C HIV-1 infected patient

**DOI:** 10.1186/1742-4690-9-S2-P291

**Published:** 2012-09-13

**Authors:** R Kumar, R Andrabi, A Tiwari, P Somi Sankaran, N Wig, D Dutta, A Sankhyan, L Khan, S Sinha, K Luthra

**Affiliations:** 1 All India Institute of Medical Sciences (AIIMS), New Delhi, India

## Background

Till date, few broadly neutralizing antibodies are generated against HIV-1 and they have limited breadth and potency against clade C viruses, which are predominant worldwide and in India. Here we have produced nine different human scFvs against the V3 region of HIV-1 envelope.

## Methods

A V3 specific phage library was constructed from EBV transformed B cells of a clade C HIV-1 infected Indian patient, whose plasma exhibited good neutralization potential against a panel of viruses. Diversity of the constructed phage library was analysed by DNA fingerprinting of 10 randomly selected clones from the unselected library using BstN1 enzyme. One round of biopanning was done against HIV-1 consensus V3C and V3B peptides. scFvs were than characterised for their binding, specifity and expression profile. VH and VL genes of anti-V3 scFvs were sequenced for their preferential gene usage.

## Results

DNA fingerprinting analysis of clones from unselected library showed that 90% of clones in the library were distinct. Thirty clones were randomly selected after biopanning. Nine clones showed binding in phage ELISA and exhibited a unique DNA fingerprint. Soluble expression of the selected scFvs was checked by SDS-PAGE and confirmed by Western blot. All the nine anti-V3 scFvs showed cross-reactivity against both the V3 peptides and did not bind to unrelated peptides. Distribution of VH gene segments of these anti-V3 scFvs were different, 56% (5/9) of scFvs used VH4, thirty 33% (3/9) VH5 and 11% (1/9) showed VH3 gene usage. Among the light chains, IGKV1 and IGKV3 were most preferentially used gene segments. Further these scFvs displayed a stable binding to V3 peptides in different denaturing agents.

## Conclusion

This is the first study to generate human anti-V3 scFvs against HIV-1 clade C. Further characterization of these scFvs for their neutralization potential and epitope mapping will provide useful information for immunogen design.

